# Upper Urinary Tract Carcinoma With Primary Small Cell Neuroendocrine Cancer—Case Report and Review of Literature

**DOI:** 10.1002/iju5.70071

**Published:** 2026-08-05

**Authors:** Siddhant Kumar, Sunil Pillai Bhaskara, A. V. B. Krishnakanth, Abhijit Shah, Siddalingeshwara Chandragouda Doddamani, Padmaraj hegde, Arun Chawla

**Affiliations:** ^1^ Department of Urology, Kasturba medical college Manipal academy of Higher education Manipal Karnataka India; ^2^ Department of Urology, Kasturba Medical college Manipal academy of Higher education Manipal Karnataka India

**Keywords:** case report, mixed histotype, renal pelvic, small cell, upper tract

## Abstract

**Introduction:**

Upper Urinary Tract Small Cell Neuroendocrine Ca are extremely rare carcinomas with less than 40 reported cases in literature.

**Case:**

92 years old male with left flank pain and diagnosed to have an enhancing renal pelvic mass, which on histopathological examination was noted to have a predominant small cell carcinoma component.

**Conclusion:**

This case report attempts to shed a light on the patients with small cell neuroendocrine carcinoma of the upper tract with its silent and aggressive nature.

**Abbreviations**
HUN: hydrouretronephrosis
IHC: immunohistochemistry
POD: post operative day
PUJ: pelviureteric junction
RGP: retrograde pyelography
SCC: small cell cancer
UUT‐SCC: upper urinary tract‐small cell cancer

**Summary**

- Upper urinary tract small cell neuroendocrine carcinomas are aggressive tumors with poor prognosis that require multimodality treatment.
- This case report attempts to bring to light a rare histopathology, its possible origin and modalities of treatment available.
- A review of available literature has been added in a concise manner.

## 1 Background

Small cell carcinomas are predominantly lung malignancies; extra pulmonary small cell carcinomas generally have an aggressive course with high chances of recurrence.

**FIGURE 1:**
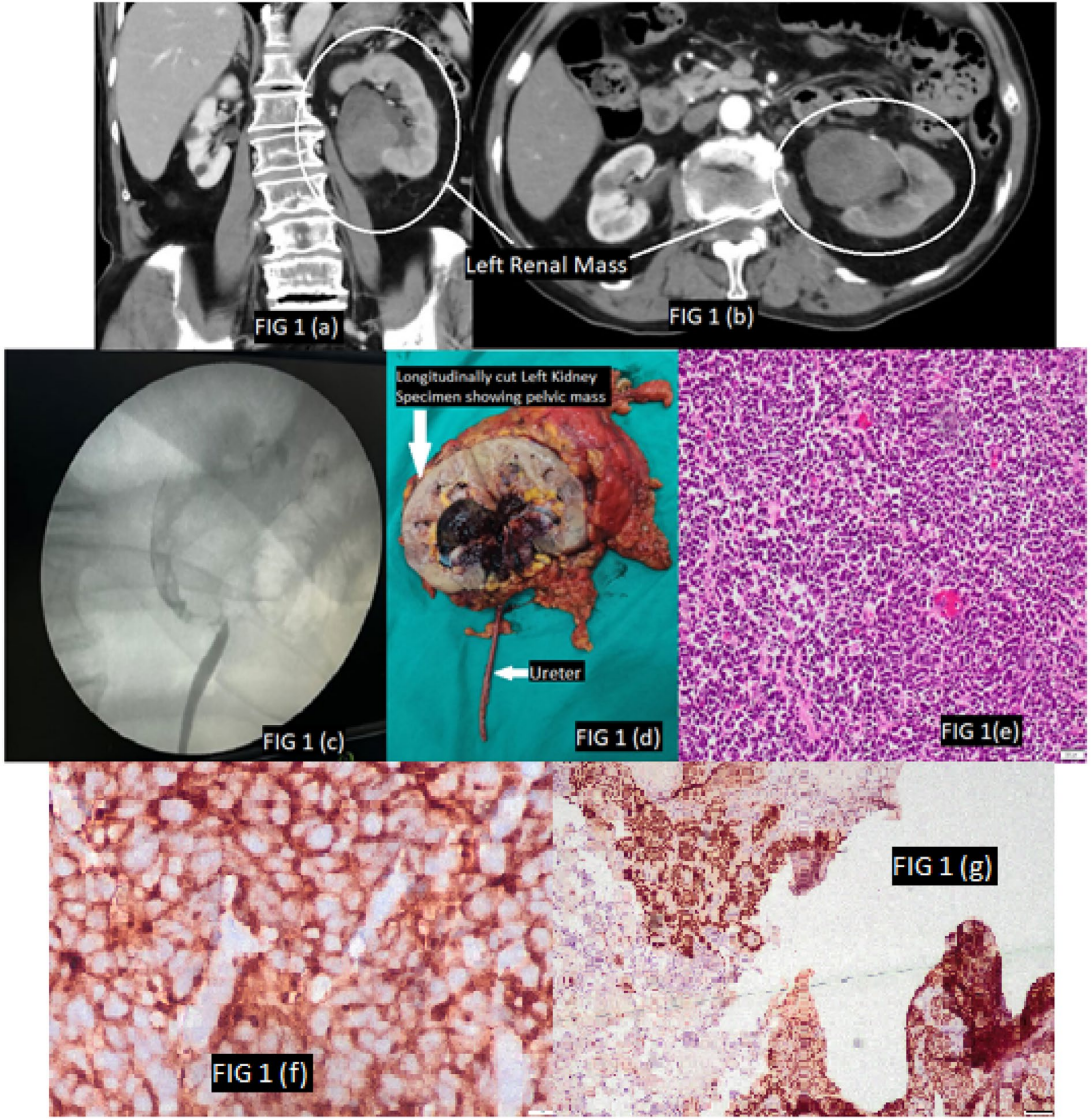
(a and b) Contrast Enhanced CT Scan showing Left Renal Pelvic Mass, (c) Left RGP showing filling defect, (d) Radical Nephroureterectomy specimen showing longitudinally cut kidney with pelvic mass, (e) HPE suggestive of Neuroendocrine tumor. (f) IHC of Small Cell Ca—showing positivity with Synaptophysin, (g) IHC of Small Urothelial Ca showing GATA 3 positivity.

SCC in the genitourinary tract is usually associated with bladder masses; with less than forty cases documented of the tumor in upper tracts [1]. Due to its rarity—their treatment poses a challenge. We therefore aim to shed light on this variant and review the literature for appropriate treatment modalities Figure 1.

## 2 Case

92 year old hypertensive male presented to our centre with left flank pain, colicky in nature and radiating to back for the last 10 days. Patient was evaluated elsewhere with ultrasonography which was suggestive of left renal mass. Patient had no history of hematuria/fever/past urological intervention. Patient was not a known smoker or drinker.

Patient was evaluated with CT Urogram, which was s/o heterogeneously enhancing soft tissue mass of 6.4 × 4.3 × 3.8 cm in the renal pelvis with mild HUN and subcentimetric lymph nodes in paraaortic region. Chest radiography did not show any primary or metastatic lesion in the lung.

Urine cytology was negative.

Cystoscopic examination showed no concurrent bladder malignancy. Left RGP showed a filling defect noted in the renal pelvis. Open Left Nephrouterectomy was done for the patient. Grossly, the renal pelvis was bulky with a soft tissue mass noted on palpation. On post‐operative day 3, the patient felt restless and was found to be tachycardic; a cardiology opinion was sought, and the patient was managed conservatively. The patient was discharged on POD 5 with stable vitals.

HPE was suggestive of Small Cell Neuroendocrine Ca (85%) with High Grade Invasive Papillary Urothelial Ca (10%–15%) and focal Squamous Differentiation (< 5%). Tumor was noted to be invading into peripelvic and perinephric fat. All margins were noted to be free of malignancy. Hilar lymph node included in the sample was found to be positive for metastasis with associated extra nodal extension and lymphovascular invasion. IHC markers were done that showed GATA 3—negative, Synaptophysin—moderate to strongly positive, and negative PAX 8.

pTNM grading of pT3N1 was made. (AJCC 8th edition).

Patient was given 4 cycles of Etoposide and Cisplatin (at 100 mg/m^2^ and 80 mg/m^2^ respectively) as adjuvant chemotherapy after multidisciplinary team consultation and is currently on follow‐up with medical oncology.

## 3 Discussion

Upper urinary tract tumors are predominantly urothelial malignancies with an incidence of approximately 1–4 cases per 1,00000 per year [2]. Neuro‐endocrine tumors include small cell ca, carcinoid tumor, and large cell ca; they account for less than 0.5% of all urinary tract tumors [3].

According to the systematic review on UUT‐SCC by Ouzzane et al., the median age of presentation for these patients was 66.5 years, and a male to female ratio of 2:1 was noted. The patients were mostly Asian (59%); they presented with complaints of most commonly hematuria. UUT‐SCC was twice as likely to be ureteral than pelvicalyceal [1].

Our patient was a 92 year old Indian‐ Asian male who presented with flank pain and a PUJ mass.

- There are 2 theories that are proposed for the histogenesis of SCC in the urinary tract—Malignant transformation of the neuroendocrine cells that are physiologically located in the urothelium.
- Arising from multipotent stem cells of urothelium [4].

As noted in our case where the histology was of a mixed type with concurrent small cell, papillary, and squamous differentiations—the theory of SCC arising from multipotent stem cells of urothelium appears more likely.

IHC markers can be used for confirmation of neuroendocrine tumors such as chromogranin—A, Synaptophysin, and neuron specific enolase [5]. In our case, Synaptophysin was positive.

In the review of literature by Ouzzane—74% of patients had locally advanced disease or malignant disease at the time of diagnosis. The same is corroborated by our case, where the patient was diagnosed to have pT3N1 disease (with perinephric and renal hilar lymph node involvement).

The staging of Urinary tract SCC is similar to SCC lung i.e., if tumor can be encompassed within 1 radiation therapy port [6]. As in our case, the tumor had extended beyond 1 radiation port, it was termed to be an extensive disease.

This emphasizes the aggressive behavior and late clinical presentation of this variant of tumors. The prognosis of patients with UUT‐SCC is poor with an overall 3‐year survival rate of 23.8% in literature [1].

Adjuvant platinum‐based chemotherapy has been shown to increase median survival by 51% as demonstrated by the POUT trial [7].

## 4 Conclusion

UUT‐SCC is an extremely rare disease with most patients presenting with advanced stages. There is a need for multimodality treatment with surgery and systemic chemotherapy. There is a need for a standardized approach to be developed.

## 5 Limitation

This being a case report has a limited scope in generalization; there is a requirement of an extensive case series or a meta analysis to help standardize treatment in patients in whom a diagnosis of small cell UTUC is made for appropriate care.

## Ethics Statement

The authors have nothing to report.

## Consent

Patient and his attendant gave consent for publication of their case report.

## Conflicts of Interest

The authors declare no conflicts of interest.
